# Supplementary material for: Cohort study of high-intensity focused ultrasound in the treatment of localised prostate cancer treatment: Medium-term results from a single centre
Source: PLoS One. 2020 Jul 23;15(7):e0236026. doi: 10.1371/journal.pone.0236026 (PMC7377399; doi:10.1371/journal.pone.0236026)
Supplement: Attachment — Submitted filename: Review comments.docx [file pone.0236026.s001.docx]

1. The major concern on this works is that it is supported the manuscript a small number of patients and a very short period in this manuscript.

2. Abstract-too much space is spent on descriptive data and not enough space on the main result of the study.

3. The author mentions in materials and methods that 405 patients with prostate cancer have been treated with HIFU from December 2009 to February 2015. From December 2009 to February 2015, 161 patients who were newly diagnosed with prostate cancer were enrolled. In total, 128 patients were included (Figure 1). It is not cleared why the author selected only 128 patients in this study and Fig.1 is confusing to understand.

4. A small group of population main concern.

Out of 161, only 128 enrolled patients were included. Not clear about the missing population in this study. Is it due to side effects? 20 patients were

excluded because of their follow-up durations were less than 30 months (not clear about missing data during that time and follow up).

Excluded 28 of 128 patients who were undergoing ADT during follow-up (Figure 1)??

Two patients were lost to follow-up (not clear)??

A very small group of studies to compare: Of the remaining 98 patients, 60 (61.2%) patients had no biochemical recurrence and thus did not require any salvage treatment, and 38 (38.8%), patients received salvage treatment due to biochemical recurrence.

Conclusion: Large group of the population required for efficacy and safety of the treatment. Clarification required for lost to follow up.

5. Prostate-specific antigen: It is very important to know the PSA levels for prostate cancer patients. So, all patients had PSA levels measured three to six months for the first few years to check how well the HIFU and salvage treatment has worked. So, I would recommend a Table including prostate-specific antigen and histology after high intensity focused ultrasound.

6. MRI analysis: MRI may provide a more sensitive test than PSA, as it can detect disease not elevating PSA but causing a change in the MRI features of residual prostatic tissue. Second, when the disease is detected on MRI, imaging also provides the location of the disease and therefore has the added advantage of being able to guide biopsy and salvage therapy by MRI analysis and the image need to include in the picture.

7. The author showed that patients with the high-risk group had shorter BCR and salvage treatment-free survival than prostate cancer patients with a low and intermediate group (P<0.0001) ([Figure 2](https://www.ncbi.nlm.nih.gov/pmc/articles/PMC4136935/figure/f2-medscimonit-20-1363/) and Figure 3). To determine whether the high-risk group has independent prognostic value, univariate and multivariate analysis needs to be performed.

8. I would recommend providing a table of Kaplan-Meier analysis of metastasis-free survival in Figure 4. What is the probability? How do certain personal, behavioral or clinical characteristics chances of survival?
