# Supplementary material for: Cohort study of high-intensity focused ultrasound in the treatment of localised prostate cancer treatment: Medium-term results from a single centre
Source: PLoS One. 2020 Jul 23;15(7):e0236026. doi: 10.1371/journal.pone.0236026 (PMC7377399; doi:10.1371/journal.pone.0236026)
Supplement: Attachment — Submitted filename: Response to Reviewers.docx [file pone.0236026.s002.docx]

Dear editor and reviewers:

Thanks for the time and effort you spent assessing the previous version of the manuscript.

For the previous table 3 (table 7 in the revised manuscript), the IIEF questionnaire was not done for most patients in that time frame, so we typed “N/A” for de novo erectile dysfunction. The column was deleted for irrelevance to the cohort of this article as reviewer’s suggestion. Other responses to the comments are as below and all of your suggestions are incorporated into revised manuscript.

Reviewer #1: The authors provided medium term oncological outcomes of a single institution cohort of 128 men receiving whole gland HIFU for clinically localized prostate cancer.

1. **I would specify that the study describes whole gland HIFU. Modify and introduce an abbreviation for whole gland HIFU throughout the manuscript.**

🡪 Thanks for this suggestion. Whole-gland HIFU may be abbreviated as WG-HIFU. It was revised in the manuscript.

2. **Abstract does not read well. For example, the first proposition does not fit neither with the background nor with the objective of the study. No information are given regarding the efficacy of salvage treatments. Please address this point.**

🡪 The abstract was revised according to your suggestion. Thanks!

3. **Did all patients received a post whole gland HIFU biopsy? Please clarify**

🡪 Post-HIFU prostate biopsy was not routinely arranged. It might be arranged because of biochemical recurrence. It might also be arranged for patients not meeting criteria of biochemical recurrence but worry about continuous elevation of PSA. We will add this paragraph to manuscript (p5, line 122-125).

4. **A table (or a Kaplan meier in case the follow up biopsy protocol was not standardized) showing the results of the follow up biopsy would be useful.**

🡪 Tables 4 and 5 showing the results of post-HIFU biopsy was added to the paragraph. Thanks for the suggestion.

5. **Which were the criteria, if there were any, to choose the salvage treatment strategy? Please describe.**

🡪 Of patients with residual tumor noted, secondary HIFU will be arranged. Of patients with local lymph node metastasis suspected, salvage radiotherapy will be suggested. Salvage radiotherapy with half to one year of ADT is the standard strategy. We will add this paragraph to manuscript (p6, line 130-134).

6. **If the authors are keen on reporting data regarding the efficacy of salvage treatments is necessary to report also the outcomes of the population receiving any salvage treatment (as subgroup analysis and figures as supplementary)**

🡪 Thanks for the suggestion. Experience of post-HIFU salvage treatment is relatively scarce. Therefore, we think we could try to share our experience after years of HIFU use. However, only 38 patients received salvage HIFU or salvage radiation therapy in this study. Their outcome was shown in figure 1. We also added some explanation about salvage treatment.

7. **Since all men received a pre mpMRI, is it possible to add any information regarding the presence of visible lesions, PI-RADS score etc?**

🡪 Thanks for this suggestion. Actually, of all 128 patients enrolled, only 12 patients received pre-HIFU mpMRI and the rest 116 patients received pre-HIFU pelvic CT. Since the number of mpMRI is small in this series, we are afraid that we cannot provide any new information about this issue.

8. **Regarding the functional outcomes, which represent secondary outcomes according to the study’s methods, only data regarding the study population should be reported. All information regarding not included cohort should be removed since not relevant.**

🡪 The irrelevant sentences and table were removed (p15). Thanks!

9. **Unless preop IIEF is available, any data regarding post op IIEF should be removed since not reliable.**

🡪 Preoperative IIEF was added to the paragraph (p15, line 232-233). Thanks for the suggestion.

10. **Adverse events should be put in one single table and, if possible, compared for each procedure (provide a p value if possible)**

🡪 The table regarding adverse events was revised and shown in table 8.

11. **The first sentence of the discussion part is out of context and biased. Please change the beginning of this paragraph**

🡪 Thanks for the reminder. This paragragh was revised (p16, line 261-266).

Reviewer #2:

1. **The major concern on this works is that it is supported the manuscript a small number of patients and a very short period in this manuscript.**

🡪 Thanks for the comment. This is a median 5-year follow-up study. We share our HIFU experience because there are only a few papers of HIFU treatment of prostate cancer.

2. **Abstract-too much space is spent on descriptive data and not enough space on the main result of the study.**

🡪 The abstract was revised. Thanks for the suggestion!

3. **The author mentions in materials and methods that 405 patients with prostate cancer have been treated with HIFU from December 2009 to February 2015. From December 2009 to February 2015, 161 patients who were newly diagnosed with prostate cancer were enrolled. In total, 128 patients were included (Figure 1). It is not cleared why the author selected only 128 patients in this study and Fig.1 is confusing to understand.**

🡪 We are sorry about the misunderstanding. In our institution, we performed the first HIFU in December 2009. From December 2009 to December 2019, when we submitted the original manuscript, there are 405 patients treated with HIFU. With the concern of adequate follow-up period, we only enrolled 161 patients treated with HIFU during December 2009 to February 2015.

4. **A small group of population main concern.**

**Out of 161, only 128 enrolled patients were included. Not clear about the missing population in this study. Is it due to side effects? 20 patients were**

**excluded because of their follow-up durations were less than 30 months (not clear about missing data during that time and follow up).**

**Excluded 28 of 128 patients who were undergoing ADT during follow-up (Figure 1)??**

**Two patients were lost to follow-up (not clear)??**

**A very small group of studies to compare: Of the remaining 98 patients, 60 (61.2%) patients had no biochemical recurrence and thus did not require any salvage treatment, and 38 (38.8%), patients received salvage treatment due to biochemical recurrence.**

**Conclusion: Large group of the population required for efficacy and safety of the treatment. Clarification required for lost to follow up.**

🡪 Of the 128 patients, 28 patients received ADT after primary HIFU as a role of salvage treatment because of biochemical recurrence or slight elevation of PSA without biochemical recurrence. Ten of the 28 patients stopped ADT months later and no more PSA elevation occurred. Eleven of the 28 patients still receive ADT currently and PSA level remain stable. Seven of the 28 patients still receive ADT currently, but gradual elevation of PSA is noted. However, they refuse or hesitate about further salvage treatment such as radiation therapy or secondary HIFU.

Since 41 of 45 (91%) suffered from biochemical recurrence within 30 months during follow-up, therefore, we excluded patients whose follow-up duration is less than 30 months to avoid missing biochemical recurrence.

5. **Prostate-specific antigen: It is very important to know the PSA levels for prostate cancer patients. So, all patients had PSA levels measured three to six months for the first few years to check how well the HIFU and salvage treatment has worked. So, I would recommend a Table including prostate-specific antigen and histology after high intensity focused ultrasound.**

🡪 Thanks for the suggestion. The relationships between post-HIFU PSA and post-HIFU prostate biopsy was shown in table 6.

6. **MRI analysis: MRI may provide a more sensitive test than PSA, as it can detect disease not elevating PSA but causing a change in the MRI features of residual prostatic tissue. Second, when the disease is detected on MRI, imaging also provides the location of the disease and therefore has the added advantage of being able to guide biopsy and salvage therapy by MRI analysis and the image need to include in the picture.**

🡪 Really thanks for your suggestion. We have described this method in the discussion in the revised manuscript (p19, line 330-334).

7. **The author showed that patients with the high-risk group had shorter BCR and salvage treatment-free survival than prostate cancer patients with a low and intermediate group (P<0.0001) (**[**Figure 2**](https://www.ncbi.nlm.nih.gov/pmc/articles/PMC4136935/figure/f2-medscimonit-20-1363/) **and Figure 3). To determine whether the high-risk group has independent prognostic value, univariate and multivariate analysis needs to be performed.**

🡪 Univariate and multivariate analysis were performed and showed in table 3.

8. **I would recommend providing a table of Kaplan-Meier analysis of metastasis-free survival in Figure 4. What is the probability? How do certain personal, behavioral or clinical characteristics chances of survival?**

🡪 Associated univariate and multivariate analysis was performed and showed in table 3.
