# Supplementary material for: Cohort study of high-intensity focused ultrasound in the treatment of localised prostate cancer treatment: Medium-term results from a single centre
Source: PLoS One. 2020 Jul 23;15(7):e0236026. doi: 10.1371/journal.pone.0236026 (PMC7377399; doi:10.1371/journal.pone.0236026)
Supplement: Attachment — Submitted filename: Response to Reviewers.docx [file pone.0236026.s003.docx]

Dear editor and reviewers:

Thanks for the time and effort you spent assessing the previous version of the manuscript. The responses to the comments are as below and all of your suggestions are incorporated into revised manuscript.

Reviewer #1:

Further minor comments:

1) **Table 3: provide HRs for both univariate and multivariate in a clearer way so that the reader can understand the confounders. logHRs not required**

🡪 Table 3 was revised according to suggestion of the Biostatistics Center of our institution.

2) **Please clarify what endoscopic biopsy means**

🡪 Endoscopic biopsy means transurethral biopsy by monopolar loop.

3) **Table 5: provide both raw numbers and percentages for each figures**

🡪 In table 5, percentage was added for each figures.

4) **positive/negative of malignancies sounds pretty bad. Rewrite it.**

🡪 The terms were revised.

5) **Please be consistent with the use of the short WG-HIFU**

🡪 Whole manuscript was examined and the WG-HIFU is used consistently.
